# Supplementary material for: Clinical outcomes of immunomodulatory therapies in the management of COVID-19: A tertiary-care experience from Pakistan
Source: PLoS One. 2022 Jan 27;17(1):e0262608. doi: 10.1371/journal.pone.0262608 (PMC8794194; doi:10.1371/journal.pone.0262608)
Supplement: Attachment — Submitted filename: RESPONSE TO REVIEWER COMMENTS plos one.docx [file pone.0262608.s001.docx]

RESPONSE TO REVIEWER COMMENTS

Reviewer: 1

Comments to the Author

Please use the space provided to explain your answers to the questions above. You may also include additional comments for the author, including concerns about dual publication, research ethics, or publication ethics. (Please upload your review as an attachment if it exceeds 20,000 characters)

Comment #1: In this tertiary-care experience from Pakistan, the authors reported clinical outcomes of immunomodulatory therapies in the management of COVID-19.

All acronyms should be explained, to help the readers to understand. Please verify all acronyms.

Response 1: Thanks for the comment. We have edited to include and verify all acronym descriptions.

Comment #2:

The section conclusion should mainly report the results of the study. On the contrary, in this version it repeats the Introduction, for this reason it should be shortened.

Response #2: We have revised conclusion section as per recommendation of reviewer

Comment #3

Due to the rapid changes in this field I suggest to update some reference:

-for example the number 3 (year 2020) should be replaced with the recent paper by Patrucco et al. Pol Arch Intern Med. 2021 Sep 30;131(9):854-861.

Response #3: We have updated reference as above

Comment #4

A relevant data, not presented in this study, is the presence of comorbidities of these patients.

This is very important because several studies have shown that comorbidities did influence the prognosis (Ejaz et al. J Infect Public Health. 2020;13(12):1833-1839) while other did not (for example Novelli et al. Panminerva Med. 2021;63(1):51-61). If the authors do not have these data, they should report it as study limitation and specify the above reported concept with references.

Response #4:We have added our comorbids data and added findings to results section. We have added the above references with respect to our findings.

Comment #5

A limitation that the authors hould be added regards the restrospective design of the study with the risk of missing data.

Response #5

We have added the above to limitation as recommended.
